# Diverse forms of HIV-1 among Burmese long-distance truck drivers imply their contribution to HIV-1 cross-border transmission

**DOI:** 10.1186/1471-2334-14-463

**Published:** 2014-08-26

**Authors:** Yan-Heng Zhou, Yue-Bo Liang, Wei Pang, Wei-Hong Qin, Zhi-Hong Yao, Xin Chen, Chiyu Zhang, Yong-Tang Zheng

**Affiliations:** Key Laboratory of Animal Models and Human Disease Mechanisms of Chinese Academy of Sciences & Yunnan Province, Kunming Institute of Zoology, Chinese Academy of Sciences, Kunming, Yunnan China; Yunnan International Travel Healthcare Center, Kunming, Yunnan 650200 China; Pathogen Diagnostic Center, Institut Pasteur of Shanghai, Chinese Academy of Sciences, Shanghai, 200025 China; University of the Chinese Academy of Sciences, Beijing, 100049 China; Shaanxi Engineering & Technological Research Center for Conversation & Utilization of Regional Biological Resources, College of Life Sciences, Yan’an University, Yan’an, Shaanxi 716000 China

**Keywords:** LDTDs, HIV-1 subtype, Recombination, Transmission, China-Myanmar border

## Abstract

**Background:**

The China-Myanmar border is a particularly interesting region that has very high prevalence of and considerable diversity of HIV-1 recombinants. Due to the transient nature of their work, long-distance truck drivers (LDTDs) have a comparatively high potential to become infected with HIV-1 and further spread virus to other individuals in the area they travel within. In this study, we hypothesized that Burmese LDTDs crossing the China-Myanmar border frequently may potentially be involved in the cross-border transmission of HIV, and contribute to the extremely high prevalence of HIV-1 inter-subtype recombinants in this border region.

**Methods:**

A molecular epidemiology study was conducted among 105 Burmese LDTDs between 2008 and 2010. HIV-1 genetic fragments including *p17*, *pol*, *vif-vpr, vpr-env,* and *C2V3* were amplified and sequenced. The subtype characterization and HIV-1 transmission were determined by both phylogenetic and phylogeographic analyses.

**Results:**

Diverse forms of HIV-1, including subtypes CRF01_AE (41.9%), C (8.6%), B (4.8%), CRF02_AG (1.0%), and inter-subtype recombinants (33.3%), as well as dual infection (10.5%), were detected among the tested LDTDs. Phylogeographic analyses based on pure subtype revealed that 77.8% Burmese LDTDs acquired HIV-1 infection in Yunnan, and the others in Myanmar. Both the C-related and CRF01_AE-related recombinants from these LDTDs appeared to have close genetic relationship with those from IDUs in Myanmar and Dehong.

**Conclusions:**

Burmese LDTDs may contribute to HIV-1 transmission along the China-Myanmar border. The results may provide some new perspective for understanding the on-going generation and prevalence of HIV-1 recombinants in the border region.

**Electronic supplementary material:**

The online version of this article (doi:10.1186/1471-2334-14-463) contains supplementary material, which is available to authorized users.

## Background

Myanmar has one of the highest rates of HIV-1 in southeast Asia, with CRF01_AE and B being the predominant subtypes among both injection drug users (IDUs) and heterosexual individuals in south and central Myanmar [1–3]. In northern Myanmar, CRF01_AE, B and C-involved unique recombinant forms (URFs) were the most prevalent strains among IDUs [4]. China’s Yunnan province, which shares some 1977 kilometers of its border with Myanmar, has the highest number of HIV-1 infected individuals in China, accounting for 34.8% of national total cases as reported [5]. In Yunnan, multiple HIV-1 genotypes, including CRF01_AE, B, C, CRF07_BC, CRF08_BC, as well as various URFs, have been identified [6, 7]. Within Yunnan, Dehong prefecture along the northern borders of Myanmar has considerably high HIV-1 prevalence among IDUs, and the proportion of HIV-1 recombinants reached 86.1%, very similar to that of northern Myanmar [7–9]. Why such an extremely high prevalence of HIV-1 recombinants occurred in both sides of the China-Myanmar border remains unclear.

Ruili city in Dehong prefecture is an important port linking China and Myanmar, serving as the major gateway for trade between both sides. Drugs (in particular heroin) from the infamous “Golden Triangle” are often illegally transferred to China via this port. Along the drug trafficking routes, HIV-1 has rapidly spread among IDUs. Unsurprisingly, Ruili became the first recorded site of HIV-1 outbreak in China [10]. About 45.2% Burmese IDUs and 55.6% Chinese IDUs cross the China-Myanmar border each month to inject drugs, often with sharing needles and syringes [Dr. Lin Duo, personal communication]. This breadth of cross-border drug usage may partly explain the reason why both sides of the China-Myanmar border have similar HIV-1 epidemic and extremely high prevalence of HIV-1 recombinants.

Earlier studies supposed that HIV-1 expanded from Myanmar to Yunnan through these drug trafficking routes, but our recent phylogeographic analyses revealed that parental subtypes (e.g. B, C and CRF01_AE) of URFs_01BC circulating among Burmese IDUs were most likely transmitted from Dehong to Myanmar during 1990s [11]. Interestingly, the prevalence of CRF01_AE-related URFs was remarkably higher in northern Myanmar than in Dehong, Yunnan, China. IDUs may be a main factor fueling HIV-1 bidirectional transmission and generation of many URFs, but could not simply explain this substantial difference in HIV-1 recombination patterns between both sides of the border. Therefore, we were curious as to whether or not other groups aside from IDUs may also have contributed to the rapid mixing of HIV-1 subtypes B, C and CRF01_AE, and the generation of the extremely high prevalence of HIV-1 recombinants in the China-Myanmar border region.

Long-distance truck drivers (LDTDs) are at an increased risk of infection with HIV, since they often stay away from their hometowns for long periods of time, and therefore less frequently visit a local, monogamous sexual partner and more frequently use commercial sex workers (CSWs) and have multiple sexual partners [12, 13]. A recent study showed that 5% of Burmese LDTDs crossing the China-Myanmar border had sex with occasional sexual partners during long-distance truck driving, and some LDTDs self-reported to inject drugs. Among this 5% population, 7.1% reported that they did not use condoms when engaging in sexual activity. HIV-1 prevalence was reported to be as high as 3.5-8.8% among Burmese LDTDs in general [14, 15]. Whether these LDTDs were involved in the high prevalence of HIV-1 recombinants in the China-Myanmar border region needs to be determined.

Here, we conducted a molecular epidemiological study of HIV-1 among Burmese LDTDs, and investigated their phylogenetic relationship with strains circulating among IDUs in the China-Myanmar border region.

## Methods

### Study population and specimens

This study was conducted in accordance with the principles of the Declaration of Helsinki and relevant international regulations, and approved by the Ethics Committee of Kunming Institute of Zoology, Chinese Academy of Sciences (Approval ID: SWYX2008010). Under the support of Australia HIV/AIDS Asia Regional Program (HAARP) and Yunnan HAARP project, 105 blood specimens were collected from HIV-positive Burmese LDTDs entering and exiting Ruili port between 2008 and 2010, after they provided written informed consent. Demographic data of all participants were obtained via an interview. The plasma were separated and stored at -70°C until use. All samples were serologically determined as HIV infection by an enzyme-linked immunosorbent assay (ELISA) kit (Wantai Biological Pharmacy Enterprise Co. Ltd, Beijing, China) and confirmed by another ELISA kit (Kehua bioengineering Co. Ltd, Shanghai, China). No HIV-2 infection was found among any of the studied participants.

### HIV-1 amplification, cloning and sequencing

Five gene fragments including *p17* (520 bp), *pol* (1273 bp), *vif-vpr* (702 bp), *vpr-env* (747 bp) and *C2V3* (513 bp) were amplified from viral RNA by RT-nested PCR as described previously [4]. The amplicons were purified and sent to sequence directly without prior cloning (Invitrogen Trading, Shanghai, China). If direct sequencing failed due to the presence of numerous double peaks in the chromatogram, the amplicons were cloned into pMD19-T vectors (TaKaRa Biotechnology) and several clones were selected randomly for re-sequencing. Furthermore, if one amplified fragment of certain samples had a subtyping result different from all other four fragments, this fragment was also subjected to TA-clone and 20–25 colonies were selected randomly for sequencing.

### Phylogenetic analyses

All obtained sequences were screened by the HIV BLAST tool to confirm lack of laboratory contamination and then were aligned with the HIV-1 reference strains obtained from the Los Alamos HIV database (http://www.hiv.lanl.gov/content/sequence/NEWALIGN/align.html), using BioEdit 7.0.9 (http://www.mbio.ncsu.edu/bioedit/bioedit.html) and MUSCLE (http://www.ebi.ac.uk/Tools/msa/muscle/). Phylogenetic trees were constructed by the neighbor-joining (NJ) method with 1000 bootstrap replicates, using Kimura’s two parameter model in MEGA 5.0 (http://www.megasoftware.net/). To detect HIV-1 recombinants, bootscan analysis was performed using SimPlot 3.5.1 (http://sray.med.som.jhmi.edu/SCRoftware/simplot/).

### Phylogeographic analyses for CRF01_AE, B, C and URFs

To investigate the infection place of HIV-1 strains circulating among LDTDs, phylogeographic analyses were performed based on *p17* sequences using BEAST 1.6.2 [16, 17]. Apart from *p17* sequences obtained in this study, all available sequences with known sample year from Myanmar, China, Thailand, Vietnam, and India were also included in the analyses. The time scaled maximum clade credibility (MCC) trees were constructed for CRF01_AE, subtype B, C and URFs via the codon-based substitution model (SRD06), the uncorrelated log-normal relaxed clock model, and the constant population size. To ensure effective sample size (ESS) values >200, Markov Chain Monte Carlo (MCMC) chains for each subtype sequence set were run for 200 million generations. All trees were visualized using FigTree 1.3.1.

### Nucleotide sequence accession numbers

All the sequences reported in this paper are available in GenBank under accession numbers from KC913597 to KC914092.

## Results

### Demographics of the study population

All 105 Burmese LDTDs were males with an average age of 34.3 (range: 20–56 years). More than half of participants (58.1%) came from Mandalay, a city in central Myanmar. Sixty five participants (61.9%) self-reported the potential routes for HIV-1 infection including 59 (56.2%) via sexual contact, two (1.9%) via blood transfusion, two (1.9%) via sexual contact and/or transfusions, and two (1.9%) via sexual and/or injection drug use (IDU). None self-reported that IDU was the only risk behavior (see Additional file 1).

### Subtype characterization of HIV-1 strains circulating among Burmese LDTDs

From 105 samples, we obtained 100 (95.2%) *p17*, 86 (81.9%) *pol*, 97 (92.4%) *vif-vpr*, 85 (81.0%) *vpr-env* and 90 (85.7%) *C2V3* sequences. Because of an overlap of 156 nt between *vif-vpr* and *vpr-env* sequences, they were spliced into a 1328 nt sequence named *vif-env* when both were available for the same sample. As a result, 80 *vif-env* sequences were obtained (see Additional file 2).

**Figure 1:**
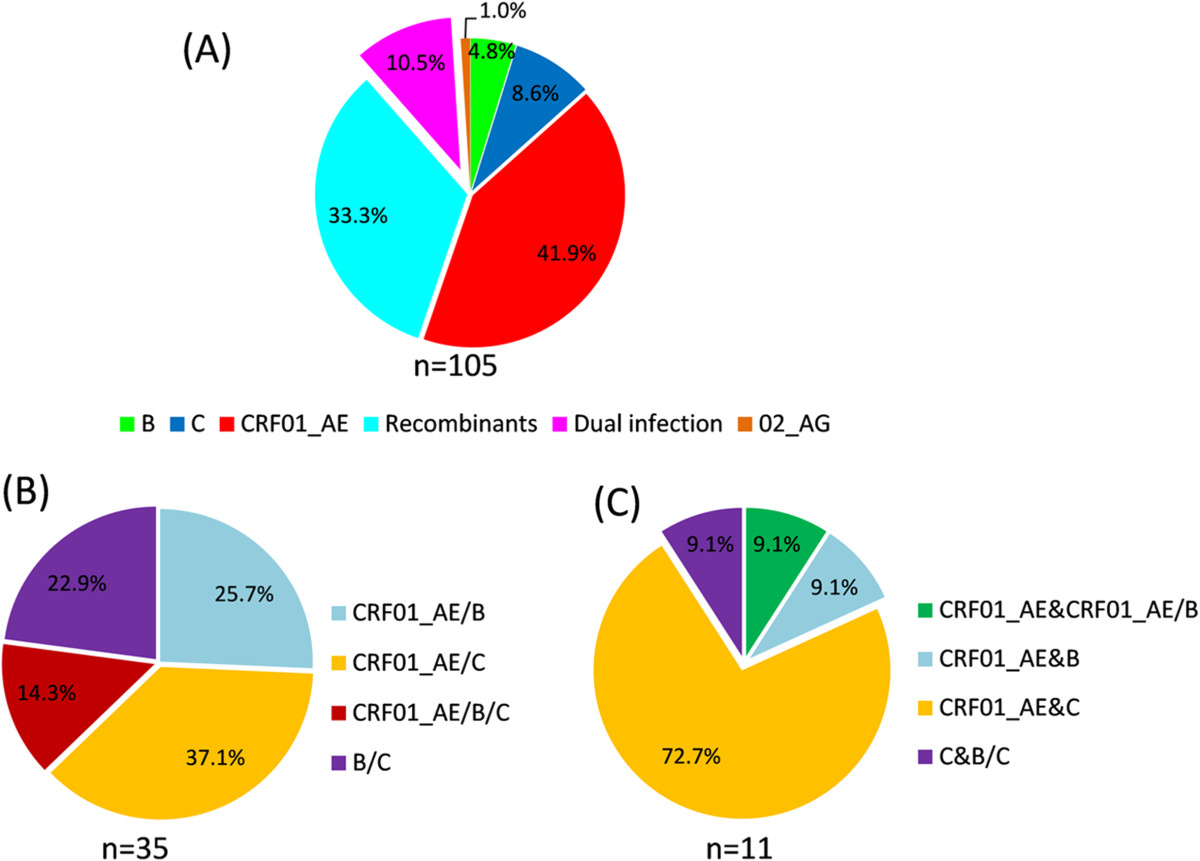
**Molecular epidemiological characterization of HIV-1 circulating among Burmese LDTDs. (A)** the proportion of HIV-1 subtypes, CRFs, recombinants and dual infection **(B)** the proportion of various recombinants **(C)** the proportion of dual infection (DI) patterns.

Based on four fragments *p17*, *pol*, *vif-env* and *C2V3*, we analyzed the subtype characterization of HIV-1 among Burmese LDTDs by constructing neighbor joining (NJ) phylogenetic trees and performing bootscan analyses. The results showed that CRF01_AE was the most dominant subtype among Burmese LDTDs, accounting for 53.5% (46/86 in *pol*) to 60.0% (60/100 in *p17*), based on different fragments. Summarizing the results of four fragments, CRF01_AE had a prevalence rate of 41.9%. Only nine C (8.6%), five B (4.8%), and one CRF02_AG (1.0%) were identified among this cohort (Figure 1A). Interestingly, a total of 35 inter-subtype recombinants were identified, accounting for 33.3%. BLAST and Simplot analyses showed that most recombinants did not share completely same recombination patterns with previously reported CRFs and URFs, suggesting that they were new URFs. The recombinants were comprised of CRF01_AE/C (37.1%), CRF01_AE/B (25.7%), B/C (22.9%), and CRF01_AE/B/C (14.3%) (Figure 1B). In details, 15 and 20 recombinants were detected based on *pol* and *vif-env* regions, respectively. Intriguingly, the strain from 08mLDTD008 has a *pol* fragment like CRF08_BC, but a *vif-env* fragment different from CRF08_BC. In addition, strains from 09mLDTD023, 09mLDTD028 and 09mLDTD029 seemed to share the same chimeric map with CRF07_BC in *vif-env* fragment, but different map in other fragments (see Additional files 2,3,4). In particular, 09mLDTD023 also shared two of three breakpoints with CRF07_BC in *pol* fragment (Additional file 3). These findings suggested that 08mLDTD008, 09mLDTD023, 09mLDTD028 and 09mLDTD029 might originate from the second-generation recombination of CRF07_BC or CRF08_BC with other subtypes.

### High prevalence of HIV-1 dual infection among LDTDs

**Figure 2:**
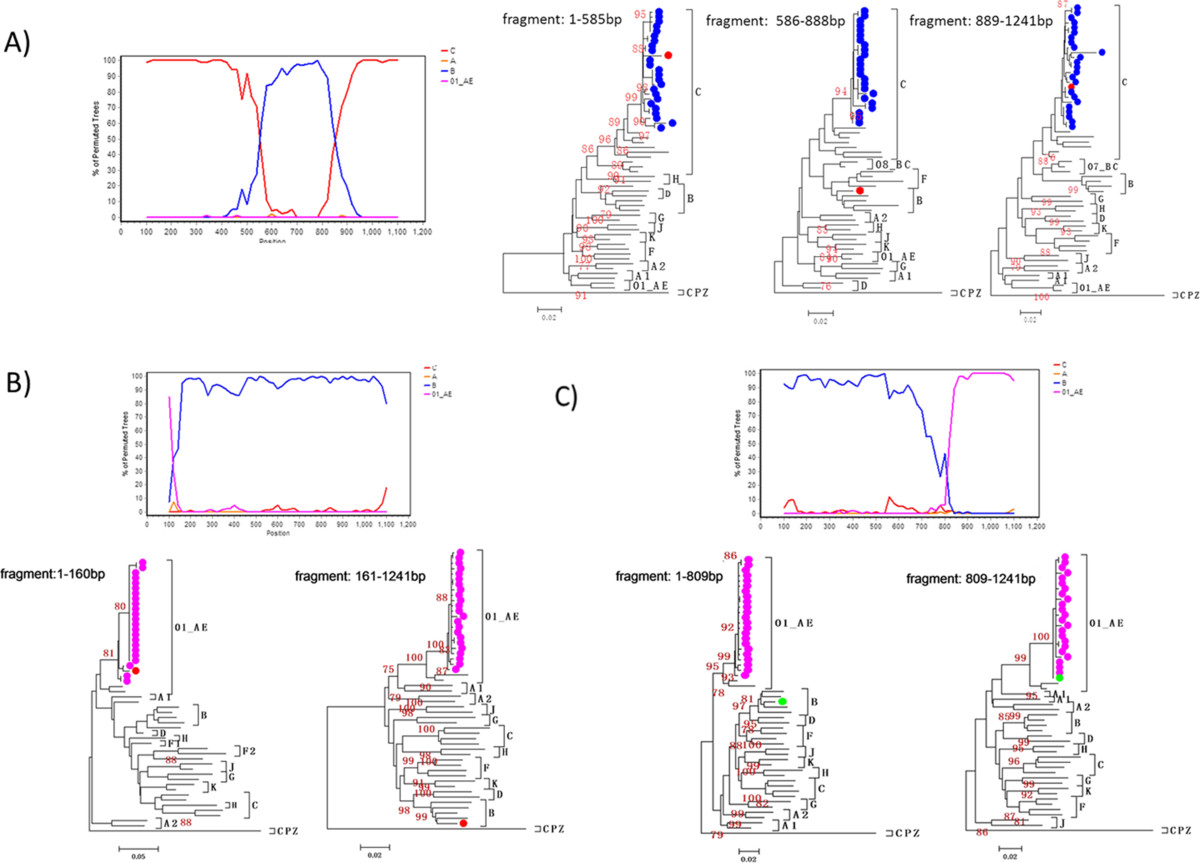
**Bootscanning plot and phylogenetic analysis of the clone sequence 08mLDTD003-1 and 09mLDTD011-23/25 based on** ***pol*** **segment. A)** the bootscan plot (left panel) and phylogenetic trees of three sub-segments (right panel) of 08mLDTD003-1 based on *pol* region. **B)** and **C)** the bootscan plots and sub-region trees for clone 09mLDTD011-23 and 09mLDTD011-25, respectively. 08mLDTD003-1 and all other clone sequences from 08mLDTD003 were highlighted by red and blue closed circle. 09mLDTD011-23, 09mLDTD011-25 and all other clone sequences from 09mLDTD011 were highlighted by red, green and purple closed circles, respectively. The subtype references for bootscan were selected as follows: A (92UG037), B’ (RL42), C (95IN21068), CRF01_AE (90CM240). The phylogenetic trees for each sub-segments were constructed by MEGA 5.0 using the neighbor-joining method, and with the clones’ sequence obtained from the same driver as well. The stability of the nodes was assessed by bootstrap analysis with 1000 replications, and only bootstrap values of >75% were shown.

Among the studied Burmese LDTDs, 10.5% were found to be dually infected with distinct subtypes or URFs. A majority (72.7%) of them were dually infected with CRF01_AE & C, followed by CRF01_AE & B (9.1%) (Figure 1C). Interestingly, 08mLDTD003 was found to be infected with subtype C and a B/C recombinant, and 09mLDTD011 was triply infected with CRF01_AE and two different CRF01_AE/B recombinants. BLAST and bootscan analyses showed that the B/C and two CRF01_AE/B recombinants mentioned above were different from any other URFs identified in this and previous studies. Further phylogenetic analyses showed that the subtype C region of the B/C recombinant from 08mLDTD003 clustered closely with the subtype C sequences from the same individual (Figure 2A). Similarly, the CRF01_AE parts of two CRF01_AE/B recombinants from 09mLDTD011 clustered closely with the CRF01_AE sequences from the same individual (bootstrap values > 80) (Figure 2B and C). Furthermore, the subtype B parts shared by the two CRF01_AE/B recombinants from 09mLDTD011 clustered together closely, suggesting a common origin of the subtype B part. Taken together, these findings suggested that the three recombinants identified in the two co-infected individuals most likely originated from recombination events recently occurring in these two individuals, rather than through direct transmission from other individuals.

### Origin of HIV-1 among LDTDs

**Figure 3:**
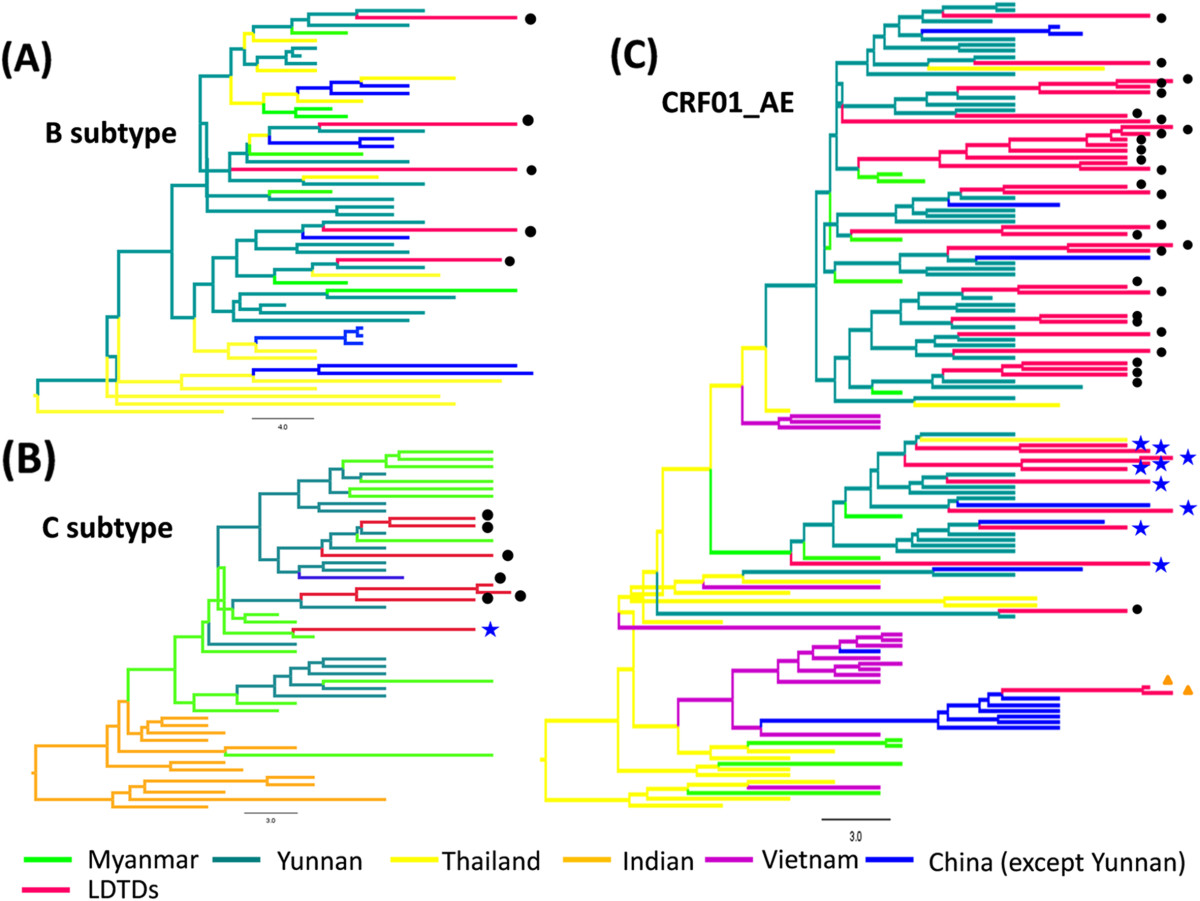
**Bayesian maximum clade credibility (MCC) trees of HIV-1 strains from Burmese LDTDs.** HIV-1 subtype B **(A)**, C **(B)** and CRF01_AE **(C)**. The trees were reconstructed based on *p17* sequences. The tree branches were signed by different color according to their respective geographical location. The black solid circle and the blue solid star indicated the sequences from Burmese LDTDs having geographic origin in Yunnan and Myanmar, respectively.

To investigate where these LDTDs might have been infected by HIV-1, phylogeographic analyses were performed based on *p17* sequences of pure subtypes. The results showed that all five subtype B (100%), six subtype C (85.7%, 6/7) and 31 CRF01_AE (73.8%, 31/42) sequences from the Burmese LDTDs had a geographic origin in Yunnan (Figure 3). Only one subtype C and 9 CRF01_AE sequences from Burmese LDTDs were found to originate in Myanmar, suggesting that Burmese LDTDs were infected with HIV-1 in either Yunnan or Myanmar.

**Figure 4:**
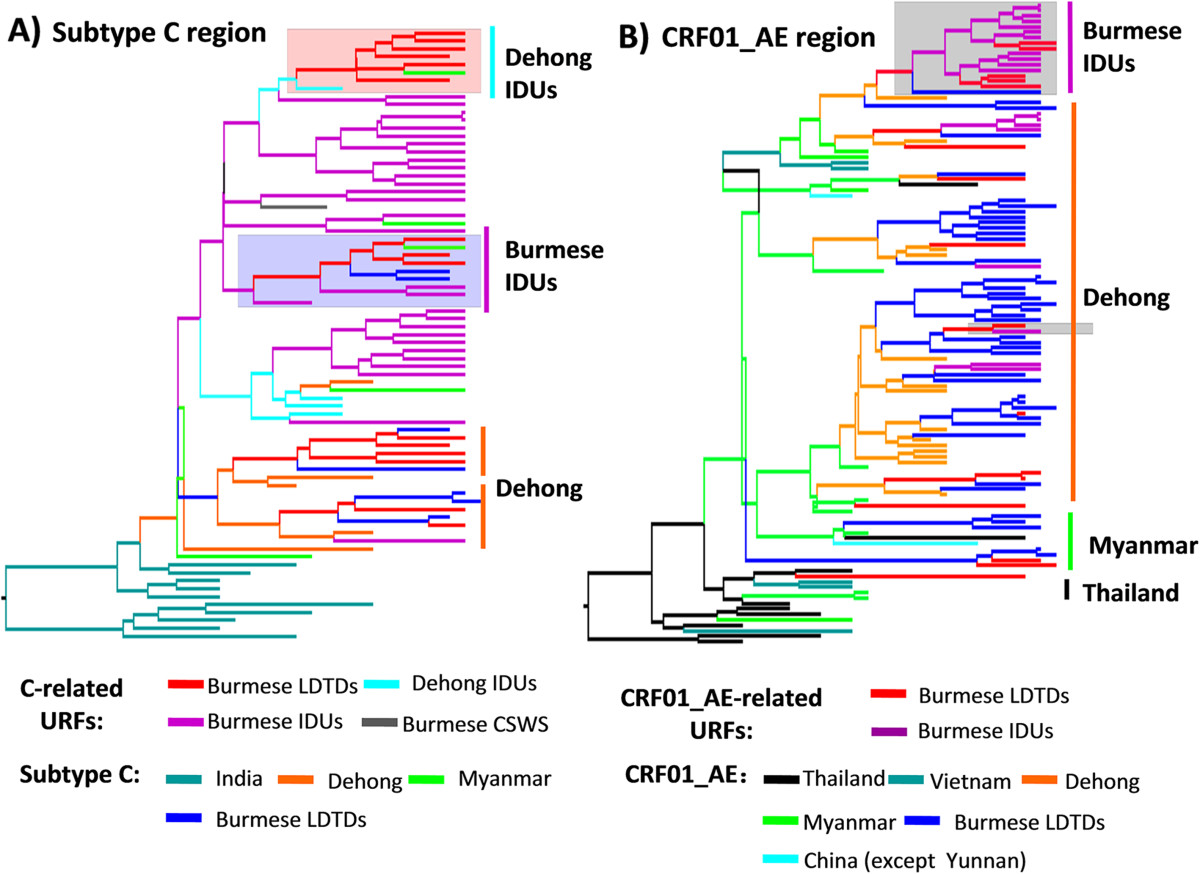
**Bayesian maximum clade credibility (MCC) trees of HIV-1 URFs from Burmese LDTDs.** *p17* fragments of HIV-1 URFs belong to subtype C **(A)** or CRF01_AE **(B)**. Trees were also reconstructed based on *p17* sequences. The reference sequences were comprised of pure subtype and the corresponding parts of the URFs from Myanmar and Dehong (download from the ref 2, 3, 4, 8). The tree branches were signed by different color according to their respective geographical location. The pink shade and lilac shade in the part A highlighted C-related URFs of Burmese LDTDs originating from Dehong IDUs and Myanmar IDUs, respectively. In the part B, the grey shade highlighted the relationship of CRF01_AE-related URFs between Myanmar IDUs and LDTDs.

In order to further investigate the potential role of LDTDs in HIV-1 transmission, we also conducted phylogeographic analyses based on *p17* regions of URFs from LDTDs, IDUs, and CSWs in Myanmar and IDUs in Dehong, China. Two MCC trees were constructed for URFs with *p17* regions of subtype C-origin (named as the C-related URFs) and CRF01_AE-origin (named as the CRF01_AE-related URFs) (Figure 4). In the tree of subtype C-related URFs, Burmese LDTDs sequences, regardless of pure subtype C sequences and the C-related URFs, clustered together and formed four clusters (Figure 4A). Two clusters including 11 Burmese LDTDs sequences and one cluster including six Burmese LDTDs sequences appeared to originate from Dehong and Dehong IDUs, respectively. The forth cluster including five Burmese LDTDs sequences appeared to have an origin from Burmese IDUs. In the tree of CRF01_AE-related URFs, majority of Burmese LDTDs sequences also clustered together. For these URFs, 5 (31.3%) originated from Dehong, 6 (40.0%) from Myanmar or Burmese IDUs, and one from Thailand. Interestingly, we found that sporadic sequences from Burmese IDUs are derived from Burmese LDTDs, suggesting that Burmese LDTDs spread HIV-1 to IDUs.

## Discussion

Population movement is commonly identified as a key factor associated with transmission of human viruses, especially HIV-1 [18]. LDTDs are a particular group with high potential to be infected with HIV-1 and further spread the virus to other individuals due to the long distances they travel, the long duration of staying away from hometowns, frequent use of CSWs, and some of them also being IDUs. Several previous studies have reported the association of the high risk behaviors of LDTDs with HIV-1 infection [15, 19]. However, there is less paper to characterize the molecular epidemiology of HIV-1 among LDTDs.

The China-Myanmar border region is a particularly interesting area that serves as a key channel for illegal drug trafficking from the “Golden triangle” to other countries/regions and has very high prevalence of HIV-1 and other viruses (e.g. HCV and HBV) [4, 9, 20]. In particular, over 86% HIV-1 strains circulating among IDUs in this region belong to URFs, which raises a question why such high proportion of HIV-1 recombinants occurred among them in this region. Furthermore, CRF01_AE-related URFs were detected among 45.6% of recombinants in northern Myanmar, significantly higher than 6.6% in its counterpart side (i.e. Dehong, Yunnan province of China) [4, 9]. IDUs were postulated as a main population responsible for the generation of so many URFs since they often injected drugs together across the border and shared needles/syringes. However, the behaviors of IDUs do not explain why such high proportion (45.6%) of CRF01_AE-related URFs occurs in northern Myanmar, implying the presence of other high-risk population responsible for this difference. In this study, we collected the samples from 105 Burmese LDTDs who drive between Mandalay, Myanmar and Ruili, China, and performed a molecular epidemiological investigation of HIV-1 among this cohort. The results showed that diverse forms of HIV-1, including subtypes CRF01_AE (41.9%), C (8.6%), B (4.8%), CRF02_AG (1.0%), and some inter-subtype recombinants (33.3%), were circulating among these LDTDs.

**Figure 5:**
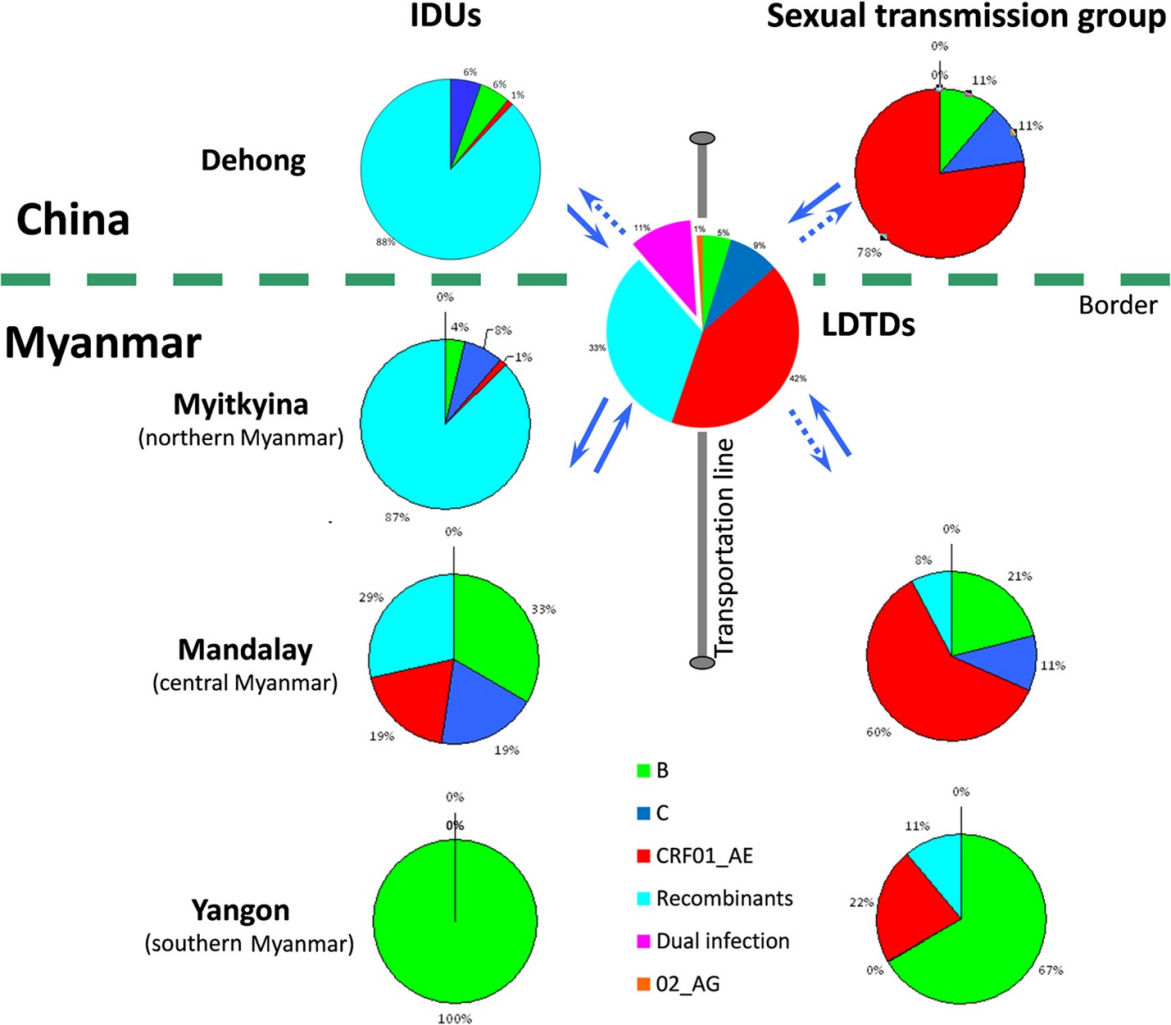
**HIV-1 subtype characterization and transmission among different high- risk groups in the China-Myanmar border area.** Data on HIV-1 subtype characterization among IDUs and sexual-infected group were taken from refs. 1, 3, 4, 6 and 9. The grey line showed the transport routes of Burmese LDTDs, which links Ruili city (in Dehong prefecture), China and Mandalay, Myanmar. The solid arrows indicated HIV-1 transmission routes obtained by phylogeographic analyses (Figures 3, 4), and the dotted arrows indicated potential HIV-1 transmission routes that need to be verified by future studies.

HIV-1 CRF01_AE is the most predominant subtype circulating among sexually acquired cases, where the prevalence of inter-subtype recombinants is relatively very low [6]. By contrast, inter-subtype recombinants are the most predominant forms circulating among IDUs, who have a very low prevalence of CRF01_AE [4, 6]. Our results showed that CRF01_AE and inter-subtype recombinants were the two most predominant forms of HIV-1 among Burmese LDTDs (Figure 1 and Additional file 2). Comparison showed that CRF01_AE prevalence among Burmese LDTDs was in the middle between heterosexuals (77.1% in Dehong and 60.5% in Mandalay) and IDUs (1.3% in Myitkyina 19.0% in Mandalay and 1% in Dehong), implying that Burmese LDTDs might contribute to the epidemiological link between heterosexuals and IDUs. Among 65 LDTDs with available information on infection routes, 59 (90.8%) self-reported that they were infected via sexual contact and two (3.1%) via sexual contact and/or IDU. This provides realistic evidence to support the role of LDTDs in linking heterosexuals and IDUs. On the other hand, the prevalence of recombinants among Burmese LDTDs was also somewhere in between both sides of the China-Myanmar border (71.4% and 87.3% in Dehong and Myitkyina IDUs, respectively) and Mandalay (28.6% and 7.9% among Mandalay IDUs and sexual intercourse group, respectively) (Figure 5). These suggest that Burmese LDTDs may also have contributed to HIV-1 transmission between China and Myanmar. Taking these results together, we speculated Burmese LDTDs might mediate the cross-border transmission of CRF01_AE from heterosexuals to IDUs.

Phylogeographic analyses of the pure subtypes showed that majority (77.8%, 42/54) of Burmese LDTDs were infected with HIV-1 (five subtype B, six subtype C and 31 CRF01_AE) in Yunnan, and the others were infected in Myanmar (one subtype C and 9 CRF01_AE) (Figure 3). Likewise, MCC trees based on *p17* regions derived from the URFs of LDTDs also supported that HIV-1 C and CRF01_AE in LDTDs have multiple origins including Dehong and Myanmar (Figure 4). On a broader scale, no matter where Burmese LDTDs acquired their infection, they have high potential to further spread the viruses to their wives and/or sexual partners in Myanmar and Yunnan. Therefore, Burmese LDTDs can mediate bi-directional transmission of HIV-1 between heterosexuals and IDUs and between China and Myanmar. This bi-directional transmission pattern of HIV-1 will facilitate the mixing of different HIV-1 forms among different high risk groups, and accelerate the generation of new recombinants, as observed among IDUs in the China-Myanmar border region. This concept was further supported by the detection of all CRF01_AE, B and C involved recombination forms (e.g. B/C, CRF01/B, CRF01/C, and CRF01/B/C) among Burmese LDTDs (Figure 1B). One major limitation is that because the samples from female sexual workers (FSWs) in the China-Myanmar border region were unavailable in this study, we were unable to obtain HIV-1 subtype characterization in this cohort, and could not find the direct evidence for the transmission association between LDTDs and FSWs. Hence we just further determined their role in HIV-1 cross-border transmission and their association with IDUs via phylogeographic analyses. To understand HIV genetic diversity and transmission complexity in the China-Myanmar border region, more concern should be paid to female sexual workers in future.

Dual infection is an essential condition for the generation of HIV-1 recombinants [21, 22]. Currently, inter-subtype recombinants are responsible for about 11.0% HIV-1 infection throughout the Asia (http://www.hiv.lanl.gov/), and dual/triple infections have been detected in Thailand and Taiwan, where multiple subtypes are co-existing [23–25]. In the China-Myanmar border area, 86.1-87.9% HIV-1 infected IDUs carried recombinants [4, 9]. Such high prevalence of recombinants implies a very frequent occurrence of dual infection. To date, however, no dual infection case was reported in this region. We found that 10.5% Burmese LDTDs were dually infected with various subtypes, implying frequent exposure of LDTDs to HIV-1. Among them, dual infection with CRF01_AE and C accounted for 72.7% (Figure 1C), which not only provided an explanation for why CRF01_AE/C recombinants were the most predominant recombination form (37.1%) among Burmese LDTDs, but also predicted an on-going generation of more new CRF01_AE/C recombinants among this population. In addition, CRF01_AE was involved in 90.9% cases of dual infection. These, together with the fact of high prevalence of CRF01_AE (41.9%), support the close association of Burmese LDTDs with sexual transmission group.Interestingly, two cases were found to be dual infection with certain pure subtype and its self-derived recombinants: one with CRF01_AE and CRF01_AE/B recombinants and the other with subtype C and B/C recombinant (Figure 2). These might represent a real-time observation on new recombinant generation. Because only the samples with sequences containing double peaks in Sanger sequencing were subjected to TA-cloning and in general, only 3–25 clones for each sample were selected for sequencing, this may lead to an underestimation of prevalence of dual infection among this cohort. The real dual infection rate may be higher when more samples are subjected to TA-cloning and sequencing, or ultra-deep sequencing, which is a robust tool to found low-frequency variants.

## Conclusions

This study firstly represented the molecular epidemiological characterization of HIV-1 among Burmese LDTDs. High prevalence of HIV-1 CRF01_AE and inter-subtype recombinants, as well as dual infection, were detected among them. Phylogeographic analyses revealed that Burmese LDTDs may have made contribution in HIV-1 transmission between the heterosexuals and IDUs and between China and Myanmar in general. These findings may provide new insight into understanding of the extensive and complex of HIV-1 recombination in the China-Myanmar border area (Figure 5). In a broader sense, our results also provide new information relevant to policy-making decisions regarding the control of HIV/AIDS in southeast Asia.

## Electronic supplementary material

Additional file 1: Characteristics and subtypes information of 105 Burmese LDTDs.(PDF 83 KB)

Additional file 2: Summary of subtype characterization of four gene fragments among 105 Burmese LDTDs.(PDF 202 KB)

Additional file 3: **Bootscanning plots of** ***pol*** **fragments of HIV-1 inter-subtype recombinants among Burmese LDTDs.** Recombination was determined using bootscan analyses (SimPlot 3.5.1 software). The HIV-1 subtype references used in bootscan analyses include subtypes A (92UG037), B’ (RL42), C (95IN21068), and CRF01_AE (90CM240). (PDF 655 KB)

Additional file 4: **Bootscanning plots of** ***vif-env*** **fragments of HIV-1 inter-subtype recombinants among Burmese LDTDs.** Because some recombinants (e.g. B/C recombinants) shared identical breakpoints, only one representative bootscanning plot was shown. For the details of reference, please see Additional file 3. (PDF 391 KB)

Below are the links to the authors’ original submitted files for images.

Authors’ original file for figure 1

Authors’ original file for figure 2

Authors’ original file for figure 3

Authors’ original file for figure 4

Authors’ original file for figure 5
