# Supplementary material for: Diverse forms of HIV-1 among Burmese long-distance truck drivers imply their contribution to HIV-1 cross-border transmission
Source: BMC Infect Dis. 2014 Aug 26;14:463. doi: 10.1186/1471-2334-14-463 (PMC4152572; doi:10.1186/1471-2334-14-463)
Supplement: Supplementary file 1 — Additional file 1: Characteristics and subtypes information of 105 Burmese LDTDs.(PDF 83 KB) [file 12879_2014_3755_MOESM1_ESM.pdf]

Additional file 1. The demographic and subtype characterizations of 105 Burmese LDTDs.

Variable (n=105)	No. of LDTD (%)	HIV Subtype		
		Pure subtype (CRF01_AE/B/C)	Recombinants	Dual infection
**Residence**				
Mandalay	61(58.1)	33(23/4/6)	22	6
Yangon	4(3.8)	1(1/0/0)	3	0
Lashio	7(6.7)	2(1/1/0)	4	1
others	32(30.5)	22(19/0/3)	7	3
Unknown	1(1.0)	0(0/0/0)	0	1
**Gender**				
**Male**	105(100)	58(44/5/9)	36	11
**Female**	0	0	0	0
**Age** (years old)				
20-30	31(29.5)	16(12/0/4)	10	5
31-40	60(57.1)	35(28/4/3)	20	5
41-50	12(11.4)	6(4/0/2)	5	1
≥51	2(1.9)	1(0/1/0)	1	0
**Route of infection**				
Sexual	59(56.2)	28(23/2/3)	24	7
Transfusion	2(1.9)	2(1/0/1)	0	0
Sexual and/or transfusion	2(1.9)	2(1/1/0)	0	0
Sexual and/or IDU	2(1.9)	1(1/0/0)	0	1
Not available	40(38.1)	25(18/2/5)	12	3
