# Supplementary material for: Diverse forms of HIV-1 among Burmese long-distance truck drivers imply their contribution to HIV-1 cross-border transmission
Source: BMC Infect Dis. 2014 Aug 26;14:463. doi: 10.1186/1471-2334-14-463 (PMC4152572; doi:10.1186/1471-2334-14-463)
Supplement: Supplementary file 2 — Additional file 2: Summary of subtype characterization of four gene fragments among 105 Burmese LDTDs.(PDF 202 KB) [file 12879_2014_3755_MOESM2_ESM.pdf]

Additional file 2. The summary of subtype characterization of four gene fragments among 105 Burmese LDTDs.

Specimen	P17	pol	Vif-env	C2V3	Total
08mLDTD001	CRF01	CRF01	CRF01	CRF01	CRF01
08mLDTD002	CRF01	CRF01	CRF01	CRF01	CRF01
08mLDTD003	C	DI by C and C/B/C(25)	C	C	DI by C and C/B/C
08mLDTD004	CRF01	CRF01	CRF01	CRF01	CRF01
08mLDTD005	CRF01	CRF01	CRF01/B	CRF01	CRF01/B
08mLDTD006	CRF01	CRF01	CRF01[a]	CRF01	CRF01
08mLDTD007	CRF01	B/CRF01	CRF01/B[b]	B	CRF01/B
08mLDTD008	C	08_BC	CRF01/C	C	CRF01/B/C
08mLDTD009	CRF01	NA	C/CRF01	C	CRF01/C
08mLDTD010	CRF01	CRF01	CRF01	CRF01	CRF01
08mLDTD011	CRF01	C/B/C	C/B/C/B/01	C	CRF01/B/C
08mLDTD012	CRF01	CRF01	CRF01	CRF01	CRF01
08mLDTD013	CRF01	CRF01	CRF01	CRF01	CRF01
08mLDTD014	NA	CRF01	CRF01[a]	CRF01	CRF01
08mLDTD015	C	C	C	C	C
08mLDTD016	CRF01	CRF01/C	CRF01[a]	C	CRF01/C
08mLDTD017	CRF01	CRF01	CRF01	CRF01	CRF01
08mLDTD018	CRF01	CRF01	B/CRF01	CRF01	CRF01/B
08mLDTD019	CRF01	CRF01	CRF01	CRF01	CRF01
08mLDTD020	CRF01	NA	CRF01	CRF01	CRF01
08mLDTD021	B	NA	B	B	B
08mLDTD022	CRF01	CRF01	CRF01	B(19)	CRF01/B
08mLDTD023	CRF01	C/CRF01/B/CRF01	CRF01/C/CRF01	CRF01	CRF01/B/C
08mLDTD024	CRF01	CRF01	CRF01	CRF01/C	CRF01/C
08mLDTD025	C	B/C	C/B/C	C	B/C
08mLDTD026	C	C/B/C/B/C	C/B/C	C	B/C
08mLDTD027	C	C	C[a]	C	C
08mLDTD028	CRF01	NA	CRF01	CRF01	CRF01
08mLDTD029	CRF01	CRF01	CRF01	CRF01	CRF01
08mLDTD030	C(20)	NA	DI by B and CRF01[b](6)	NA	DI by B and CRF01
08mLDTD031	C	C	C[a]	C	C
08mLDTD032	C	C/B/C/B	C	C	B/C
08mLDTD033	DI by CRF01 and C(6)	NA	CRF01	CRF01	DI by CRF01 and C
08mLDTD034	CRF01	CRF01	CRF01	CRF01	CRF01
08mLDTD035	CRF01	CRF01	CRF01	CRF01	CRF01
08mLDTD036	CRF01	CRF01	CRF01	CRF01	CRF01
08mLDTD037	NA	CRF01	CRF01	CRF01	CRF01
08mLDTD038	C	C	C	NA	C

continued

08mLDTD039	C(5)	DI by CRF01 and C(25)	C[a]	NA	DI by CRF01 and C
08mLDTD040	CRF01	CRF01	CRF01	NA	CRF01
08mLDTD041	DI by CRF01 and C(5)	B/CRF01	CRF01/B/CRF01(3)	CRF01	DI by CRF01 and C(5)
08mLDTD042	CRF01	CRF01	CRF01	NA	CRF01
08mLDTD043	CRF01	CRF01	CRF01	CRF01	CRF01
08mLDTD044	CRF01	CRF01	CRF01	CRF01	CRF01
09mLDTD001	CRF01	CRF01	CRF01	CRF01	CRF01
09mLDTD002	DI by CRF01 and C(6)	CRF01	CRF01	C(6)	DI by CRF01 and C
09mLDTD003	CRF01	NA	C/CRF01/B/CRF01	C	CRF01/B/C
09mLDTD004	C	C/CRF01	C/CRF01	NA	CRF01/C
09mLDTD005	B	NA	B[b](5)	CRF01(20)	CRF01/B
09mLDTD006	CRF01	CRF01	CRF01	NA	CRF01
09mLDTD007	DI by CRF01 and C (6)	CRF01	CRF01[a]	NA	DI by CRF01 and C
09mLDTD008	B	B	CRF01/B	B	CRF01/B
09mLDTD009	CRF01	CRF01	CRF01	CRF01	CRF01
09mLDTD010	CRF01	CRF01	CRF01[a]	CRF01	CRF01
09mLDTD011	CRF01(6)	DI by CRF01 and CRF01/B(25)	CRF01[b]	CRF01	DI by CRF01 and CRF01/B
09mLDTD012	02_AG	NA	02_AG	02_AG	02_AG
09mLDTD013	CRF01	DI by CRF01 and C (4)	C	C	DI by CRF01 and C
09mLDTD014	C	C	C[b]	CRF01	CRF01/C
09mLDTD015	CRF01/B	CRF01/B/CRF01	CRF01	CRF01	CRF01/B
09mLDTD016	CRF01	CRF01	CRF01	CRF01	CRF01
09mLDTD017	CRF01	CRF01	CRF01	B(22)	CRF01/B
09mLDTD018	B	B	NA	B	B
09mLDTD019	C	NA	CRF01[a]	C	CRF01/C
09mLDTD020	CRF01	CRF01	CRF01	CRF01/C	CRF01/C
09mLDTD021	CRF01	NA	CRF01	CRF01	CRF01
09mLDTD022	CRF01	CRF01	CRF01	CRF01	CRF01
09mLDTD023	C	C/B/C	07_BC	C	B/C
09mLDTD024	C	C/B	C/CRF01[a]	C	CRF01/B/C
09mLDTD025	CRF01	CRF01	CRF01	CRF01	CRF01
09mLDTD026	B	B	B	B	B
09mLDTD027	CRF01	CRF01	CRF01	NA	CRF01
09mLDTD028	C	B	07_BC	NA	B/C
09mLDTD029	NA	B	07_BC	C	B/C
09mLDTD030	B	B	B	B	B
09mLDTD031	CRF01	CRF01	CRF01	CRF01	CRF01
09mLDTD032	C	NA	C/B/C/B	C	C/B/C
09mLDTD033	B	B	B[a]	NA	B

continued

09mLDTD034	CRF01	NA	CRF01	NA	CRF01
09mLDTD035	CRF01	CRF01	CRF01	CRF01	CRF01
09mLDTD036	C	C	C[a]	DI by CRF01 and C(18)	DI by CRF01 and C
09mLDTD037	CRF01	NA	CRF01	CRF01	CRF01
09mLDTD038	C	NA	C/CRF01[a]	CRF01	CRF01/C
09mLDTD039	CRF01	C/CRF01/C	CRF01	CRF01	CRF01/C
09mLDTD040	CRF01	CRF01	CRF01	CRF01	CRF01
09mLDTD041	CRF01	CRF01	CRF01[a]	CRF01	CRF01
09mLDTD042	C(6)	CRF01(22)	CRF01	C	CRF01/C
09mLDTD043	C	C	NA	C	C
09mLDTD044	CRF01	CRF01	CRF01	CRF01	CRF01
09mLDTD045	CRF01	CRF01	CRF01	CRF01	CRF01
09mLDTD046	C	C	C	C	C
10mLDTD001	C	NA	CRF01	C	CRF01/C
10mLDTD002	CRF01	C	C/CRF01	C	CRF01/C
10mLDTD003	CRF01	CRF01/B	CRF01[a]	NA	CRF01/B
10mLDTD004	CRF01	CRF01	CRF01	CRF01	CRF01
10mLDTD005	NA	C	C[a]	C	C
10mLDTD006	C	C	C	C	C
10mLDTD007	CRF01	C/CRF01/C	C/CRF01[a]	CRF01	CRF01/C
10mLDTD008	DI by C and CRF01(5)	C	C/CRF01/B	C	DI by C and CRF01
10mLDTD009	CRF01	CRF01	CRF01	CRF01	CRF01
10mLDTD010	CRF01	CRF01	CRF01	CRF01	CRF01
10mLDTD011	NA	NA	C	C	C
10mLDTD012	CRF01	NA	CRF01	NA	CRF01
10mLDTD013	CRF01	CRF01	NA	CRF01	CRF01
10mLDTD014	CRF01	NA	CRF01	CRF01	CRF01
10mLDTD015	C	B	C/B/C	NA	B/C

The number in the parenthesis represented the count of sequences obtained from clones.

DI: dual infection; CRF01: CRF01_AE; NA: not available.

a: subtyping based on vif-vpr fragment.

b: subtyping based on vpr-env fragment.
