# Supplementary material for: Diverse forms of HIV-1 among Burmese long-distance truck drivers imply their contribution to HIV-1 cross-border transmission
Source: BMC Infect Dis. 2014 Aug 26;14:463. doi: 10.1186/1471-2334-14-463 (PMC4152572; doi:10.1186/1471-2334-14-463)
Supplement: Supplementary file 3 — Additional file 3: Bootscanning plots of pol fragments of HIV-1 inter-subtype recombinants among Burmese LDTDs. Recombination was determined using bootscan analyses (SimPlot 3.5.1 software). The HIV-1 subtype references used in bootscan analyses include subtypes A (92UG037), B’ (RL42), C (95IN21068), and CRF01_AE (90CM240). (PDF 655 KB) [file 12879_2014_3755_MOESM3_ESM.pdf]

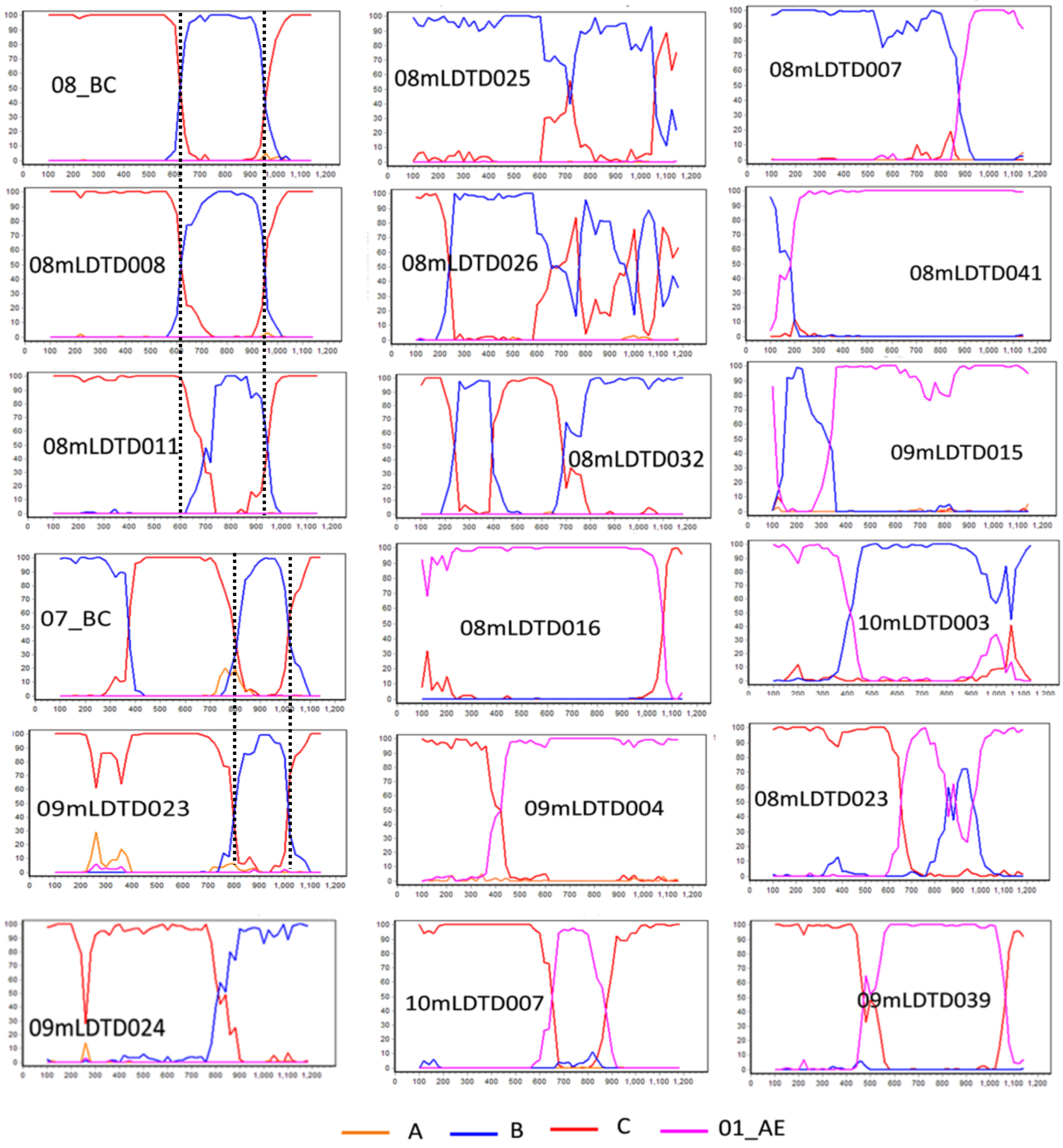

Additional file 3. Bootscanning plots of *pol* fragments of HIV-1 inter-subtype recombinants among Burmese LDTDs. The recombination was determined using bootscan analyses (SimPlot 3.5.1 software). The HIV-1 subtype references used in bootscan analyses were: subtypes A (92UG037), B' (RL42), C (95IN21068), and CRF01_AE (90CM240).
