# Supplementary material for: Diverse forms of HIV-1 among Burmese long-distance truck drivers imply their contribution to HIV-1 cross-border transmission
Source: BMC Infect Dis. 2014 Aug 26;14:463. doi: 10.1186/1471-2334-14-463 (PMC4152572; doi:10.1186/1471-2334-14-463)
Supplement: Supplementary file 4 — Additional file 4: Bootscanning plots of vif-env fragments of HIV-1 inter-subtype recombinants among Burmese LDTDs. Because some recombinants (e.g. B/C recombinants) shared identical breakpoints, only one representative bootscanning plot was shown. For the details of reference, please see Additional file 3. (PDF 391 KB) [file 12879_2014_3755_MOESM4_ESM.pdf]

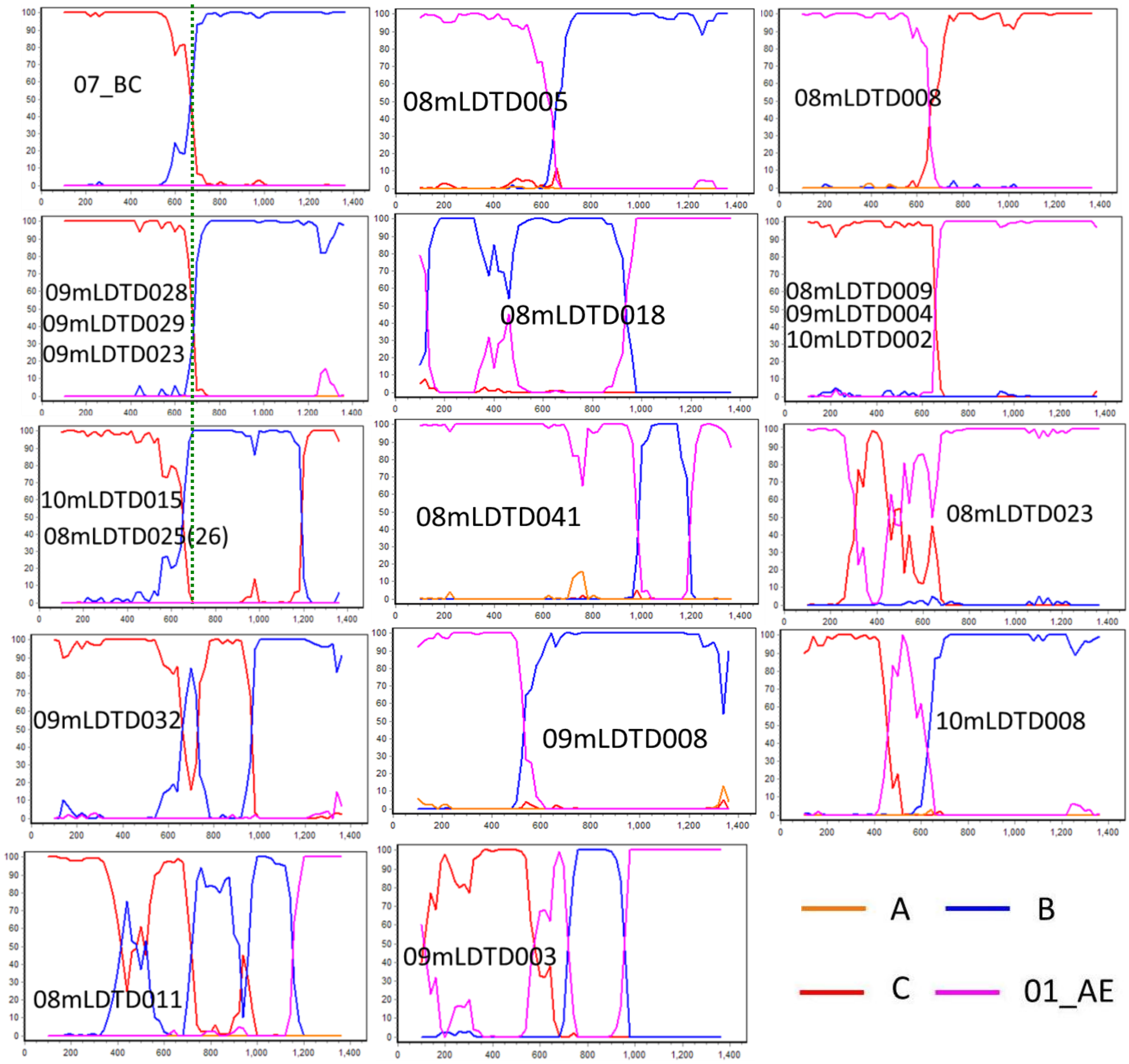

Additional file 4. Bootscanning plots of *vif-env* fragments of HIV-1 inter-subtype recombinants among Burmese LDTDs. Because some recombinants (i.e. B/C recombinants) shared identical breakpoint, only one representative bootscanning plot was shown. For the details of reference, please see Additional file 3.
