# Supplementary figures and images for: Diverse forms of HIV-1 among Burmese long-distance truck drivers imply their contribution to HIV-1 cross-border transmission
Source: BMC Infect Dis. 2014 Aug 26;14:463. doi: 10.1186/1471-2334-14-463 (PMC4152572; doi:10.1186/1471-2334-14-463)

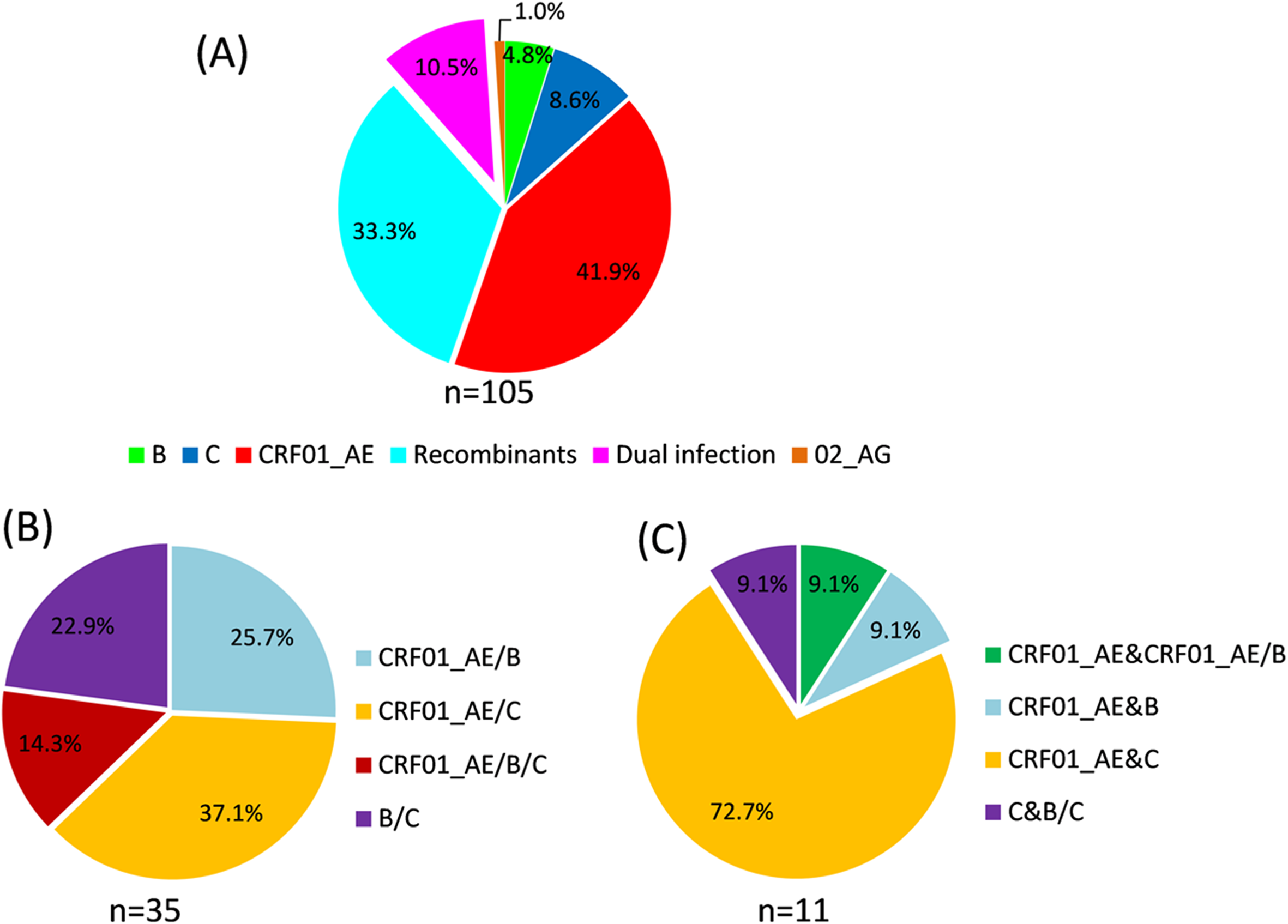

Supplement: Supplementary file 5 — Authors’ original file for figure 1 [file 12879_2014_3755_MOESM5_ESM.tif]

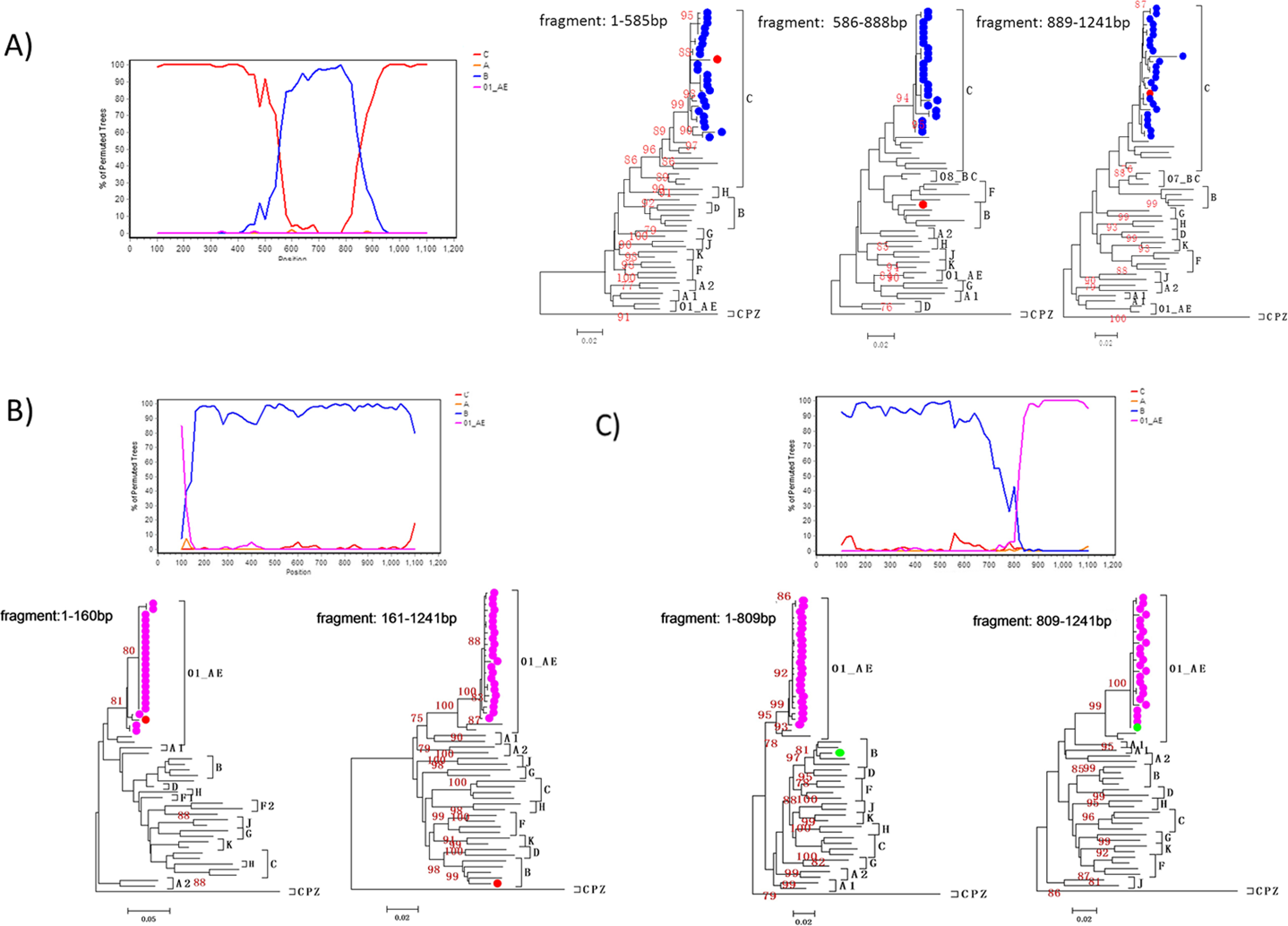

Supplement: Supplementary file 6 — Authors’ original file for figure 2 [file 12879_2014_3755_MOESM6_ESM.tif]

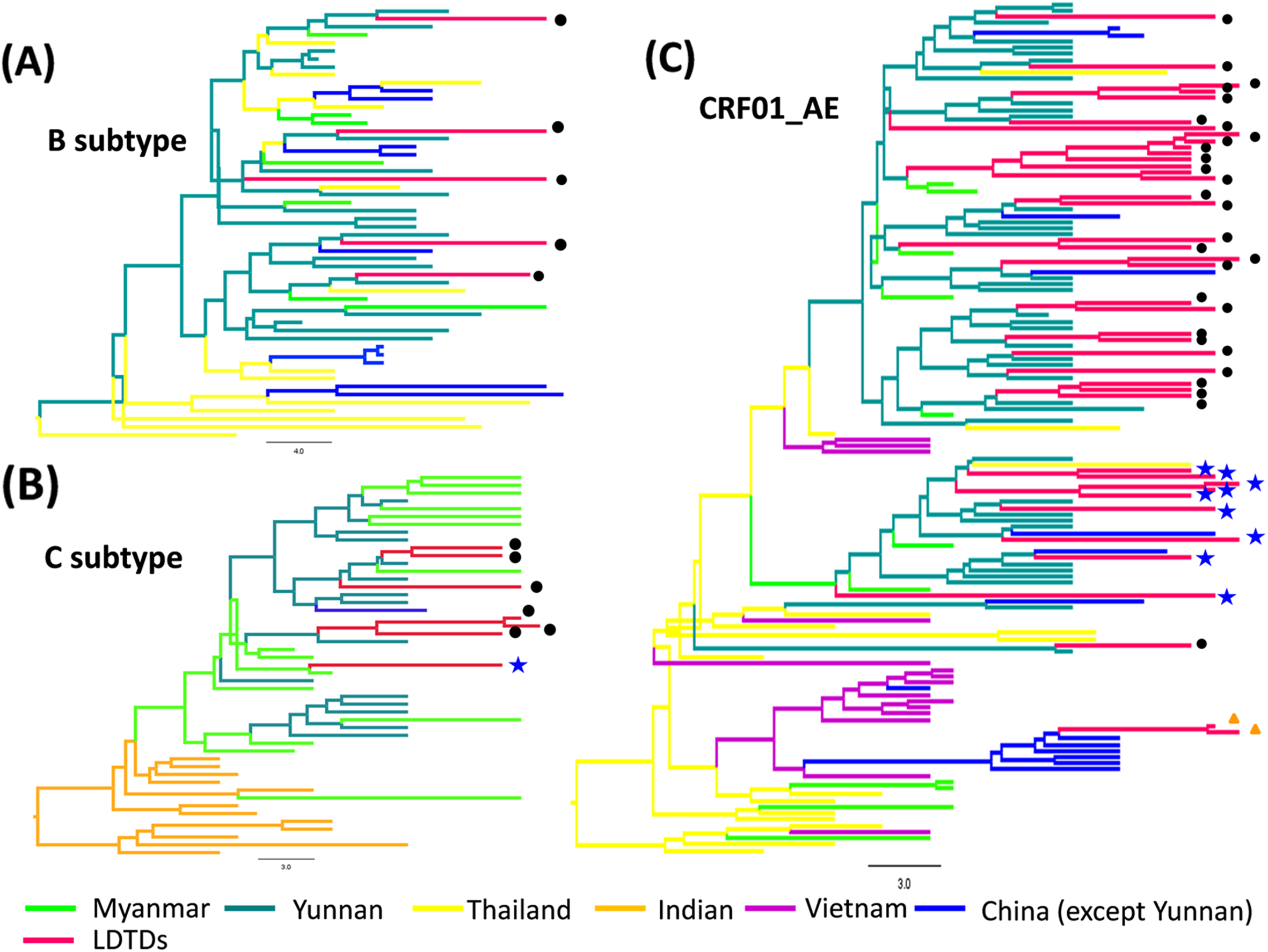

Supplement: Supplementary file 7 — Authors’ original file for figure 3 [file 12879_2014_3755_MOESM7_ESM.tif]

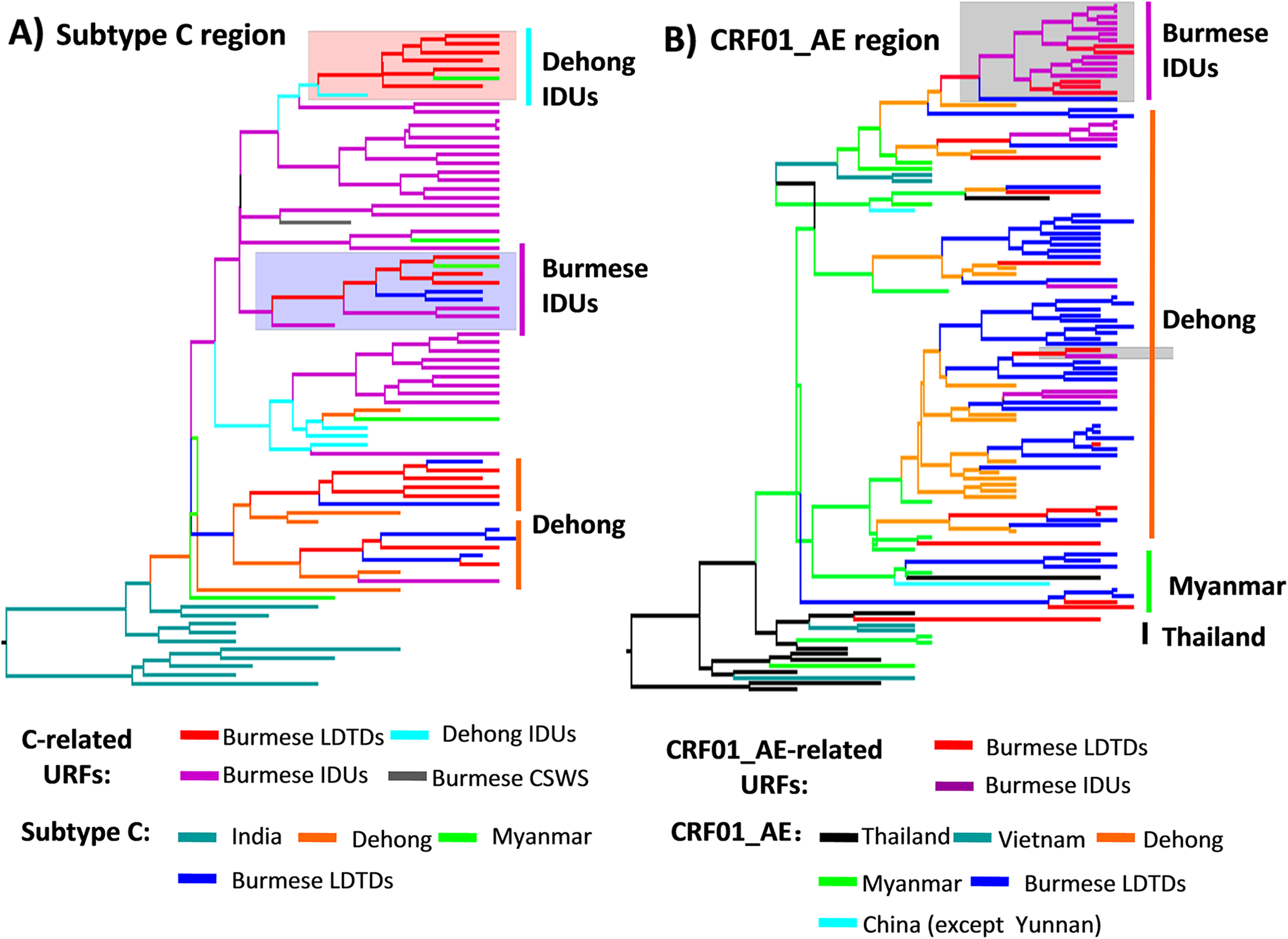

Supplement: Supplementary file 8 — Authors’ original file for figure 4 [file 12879_2014_3755_MOESM8_ESM.tif]

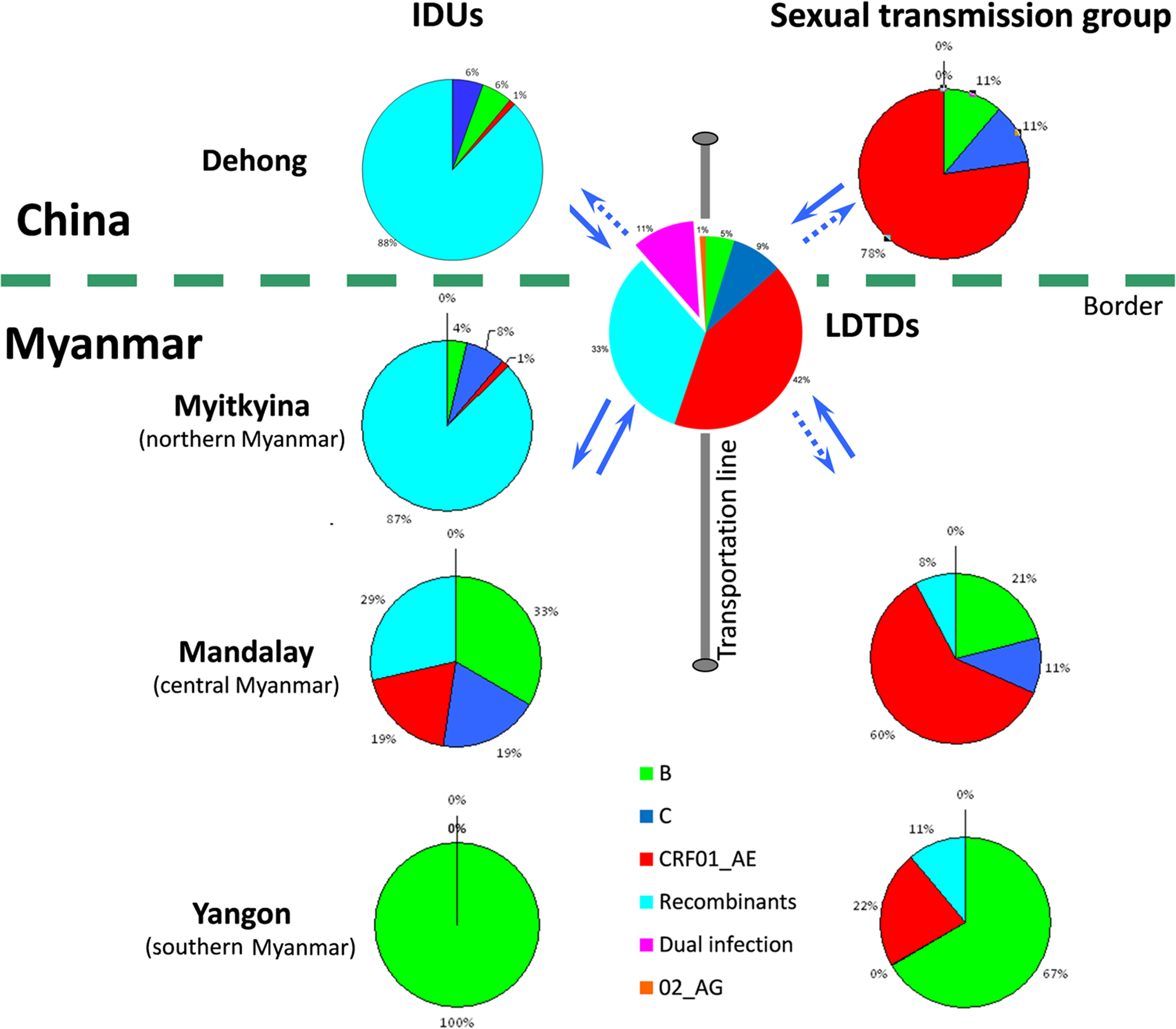

Supplement: Supplementary file 9 — Authors’ original file for figure 5 [file 12879_2014_3755_MOESM9_ESM.tif]
